# Genetic overlap between autoimmune disorders and anorexia nervosa: insights from a large-scale cross-trait genome-wide analysis

**DOI:** 10.1186/s40337-026-01635-5

**Published:** 2026-05-29

**Authors:** Xu Lin, Yu-Sheng Huang, Ya-Juan Xu, Gui-Bing Chen

**Affiliations:** 1 Xiamen Xianyue Hospital, Xianyue Hospital Affiliated with Xiamen Medical College, Fujian Psychiatric Center, Fujian Clinical Research Center for Mental Disorders, No.387~399 of Xianyue Road, Xiamen, 361000 Fujian China; 2 The School of Clinical Medicine, Fujian Medical University, Fuzhou, Fujian China

**Keywords:** Genetic overlap, Anorexia nervosa, Autoimmune disease, Mendelian randomization

## Abstract

**Background:**

To investigate the shared genetic architecture between autoimmune disorders and anorexia nervosa (AN), identify common risk loci and genes, and explore the underlying genetic mechanisms.

**Methods:**

Using summary-level data from large-scale genome-wide association studies (GWAS), we assessed genetic overlap between autoimmune disorders and AN and conducted cross-trait linkage disequilibrium score regression (LDSC) analysis to identify shared loci and genes. A series of functional annotation and tissue-specific analyses were performed to explore the effects of pleiotropic genes. Genetic enrichment analyses were conducted to identify key tissues involved. Finally, bidirectional Mendelian randomization (MR) analysis was used to investigate causal relationships.

**Results:**

Our study highlighted shared genetic mechanisms between six autoimmune diseases and AN. Through gene-based analysis of variants screened by PLACO (FDR < 0.05), 38 pleiotropic genes were identified at the genome-wide significance level (*P* < 5 × 10^-8), with strong colocalization evidence for three loci (PRKAR2A, CELSR3, and KLHDC8B). Tissue enrichment at both the SNP and gene levels revealed a crucial role for brain tissues in the pleiotropic mechanisms. Through MR analysis, we identified protective causal effects of primary sclerosing cholangitis (PSC) and Graves’ disease (GD) on AN risk.Conclusions: Our findings provide strong evidence for the shared genetic architecture between autoimmune diseases and AN and offer potential insights into the underlying mechanisms involved.

**Conclusions:**

Our findings provide strong evidence for the shared genetic architecture between autoimmune diseases and AN and offer potential insights into the underlying mechanisms involved.

**Supplementary Information:**

The online version contains supplementary material available at 10.1186/s40337-026-01635-5.

## Plain language summary

This study utilized large-scale genetic databases to investigate the shared genetic risks between anorexia nervosa and autoimmune diseases. We found significant genetic overlap between six autoimmune diseases (such as asthma, primary sclerosing cholangitis, and Graves’ disease) and anorexia nervosa, identifying 38 shared gene regions. Surprisingly, Mendelian randomization analysis indicated that having primary sclerosing cholangitis or Graves’ disease may actually reduce the risk of developing anorexia nervosa. These findings reveal unexpected genetic links between mental disorders and the immune system, providing important clues for the development of new prevention and treatment strategies in the future.

**Supplementary Information:**

The online version contains supplementary material available at 10.1186/s40337-026-01635-5.

## Introduction

Anorexia nervosa (AN) is one of the most severe psychiatric disorders, ranking as the third most common chronic illness among adolescents and exhibiting the highest morbidity and mortality rates among all mental health conditions [1, 2]. Eating disorders (EDs) primarily emerge during adolescence and predominantly affect females [3]. Effective treatment for EDs remains challenging, with limited psychological and pharmacological interventions available, and their therapeutic efficacy is often suboptimal [4–6].

Epidemiological studies have suggested a potential link between autoimmune diseases and AN. A growing body of evidence from large-scale register-based studies indicates a bidirectional relationship between EDs and a range of autoimmune diseases (e.g., type 1 diabetes, celiac disease, Crohn’s disease, rheumatoid arthritis) [7–10]. For instance, compared to healthy controls, individuals with AN exhibit evidence of altered humoral immune responses, lipid metabolism, and plasma cell activity [11]. Furthermore, the pro-inflammatory phenotype observed in AN patients has been associated with hypothalamic antigen-specific autoantibodies, with inflammatory status potentially diminishing over the course of the illness [12]. The presence of anti-hypothalamic autoantibodies in AN patients, along with reduced serum uric acid levels and decreased total antioxidant capacity (TAC), further indicates increased oxidative stress [13]. These findings underscore a significant knowledge gap in this field, highlighting the urgent need to identify common genetic risk loci underlying both conditions.

Traditional clinical or epidemiological studies often face challenges in ensuring statistical robustness when examining such associations, including potential confounding by factors such as age, sex, socioeconomic status, and surveillance bias, as well as difficulties in establishing temporal sequence and ensuring sufficient sample size for rare conditions [7, 9]. Recently, linkage disequilibrium (LD) score regression (LDSC) has been developed to assess genetic correlations between traits and diseases [14]. However, it remains unclear whether overall genetic correlations arise from a few specific loci or are dispersed across the genome. To date, few studies have systematically evaluated the genetic overlap, shared susceptibility genes, and causal relationships between autoimmune disorders and AN.

Cross-trait analysis leverages GWAS signals to investigate pleiotropic genetic variants and loci across multiple traits and has been shown to be effective in identifying shared genetic factors between diseases [15–17]. These pleiotropic loci serve as potential intervention targets, offering opportunities for simultaneous prevention or treatment of these conditions. Additionally, a novel method known as “PLACO” (pleiotropic analysis under the composite null hypothesis) has been introduced, which employs an inverse-χ2 test (IUT) to identify pleiotropic effects at the SNP level [18].

**Fig. 1:**
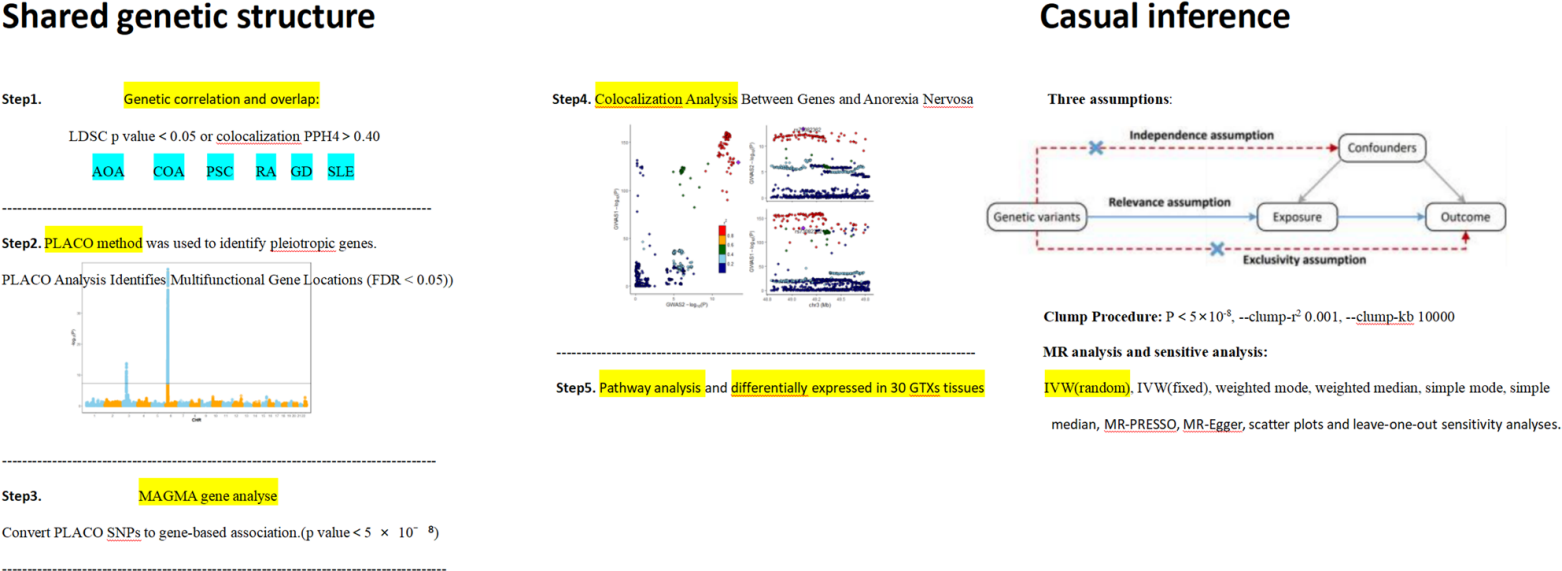
Study workflow. LDSC, linkage disequilibrium score regression; AOA, adult-onset asthma; COA, childhood-onset asthma; GD, Grave’s disease; PSC, primary sclerosing cholangitis; RA, rheumatoid arthritis; SLE, systemic lupus erythematosus; PLACO, pleiotropic analysis under the composite null hypothesis; SNPs, single nucleotide polymorphisms; MAGMA, multi-marker analysis of genomic annotation; MR, Mendelian randomisation; IVW, inverse variance weighted

Given the increasing epidemiological and biological evidence supporting the involvement of shared mechanisms (including possible genetic factors) in autoimmune disorders and AN, it is crucial to identify specific genetic variants and loci that drive genome-wide genetic correlations and to further explore the common genetic etiology of these diseases. In this regard, a recent transcriptomic study in AN patients identified significant dysregulation of genes enriched in RNA metabolism and innate immune pathways, including SNORD3C, further supporting the interplay between immune dysregulation, metabolic disturbance, and psychiatric pathology in AN [19]. A detailed flowchart outlining our study design is presented in Fig. 1.

## Research methods and materials

### Data sources

We obtained publicly available GWAS summary statistics for 16 autoimmune diseases from large-scale GWAS or meta-analyses: adult-onset asthma (AOA), childhood-onset asthma (COA) [20], Graves’ disease (GD), Hashimoto’s disease (HD) [21], hypothyroidism (HT) [22], primary biliary cirrhosis (PBC) [23], primary sclerosing cholangitis (PSC) [24], inflammatory bowel disease (IBD) [25], Crohn’s disease (CD) [26], ulcerative colitis (UC), rheumatoid arthritis (RA) [25], systemic lupus erythematosus (SLE) [21], multiple sclerosis (MS) [27], systemic sclerosis (SS) [28], type 1 diabetes (T1D) [29], and vitiligo [30].

GWAS summary statistics for AN were obtained from a meta-analysis of GWAS data comprising 16,992 cases and 55,525 controls of European ancestry [31]. Each study followed a standardized quality control procedure, and associations between case-control status and single nucleotide polymorphisms (SNPs) were assessed using logistic regression. Genetic principal components were used as covariates in the association analysis, and risk estimates were combined using a fixed-effects inverse variance weighted (IVW) meta-analysis. The sources and details of these datasets are summarized in Additional File 1: Table S1.

### Research method

#### Genetic overlap at the genome-wide level

To evaluate the genetic correlation between autoimmune disorders and AN, LDSC was used. LD scores were computed based on common SNP genotypes from European ancestry samples in the 1000 Genomes Project [32]. Standard errors (SEs) were estimated using the jackknife method to correct for attenuation bias. The LDSC intercept was used to assess potential population overlap between different datasets. The analysis confirmed that there was no significant population overlap between autoimmune disorder and AN studies.

To investigate whether autoimmune diseases and AN share causal genetic variants, we performed a region-based colocalization analysis using the coloc package (version 5.2.3) in R. Specifically, for each disease pair, we first identified the most significant lead SNP (i.e., the variant with the lowest P-value) in the base GWAS dataset. A 1-Megabase (1 MB) region of interest was then defined, centered strictly on this lead SNP (500 kb upstream and 500 kb downstream). We extracted the summary statistics for all overlapping SNPs within this defined 1 MB window from both the autoimmune disease and AN datasets. The colocalization analysis was subsequently conducted on this region using default prior probabilities (p1 = 1 × 10^− 4^, p2 = 1 × 10^− 4^, p12 = 1 × 10^− 5^). Colocalization analysis was conducted to determine whether the association between specific autoimmune disorders and AN could be attributed to the same causal genetic variants. This analysis was based on a Bayesian model, which assigns posterior probabilities to five hypotheses: H0 represents no association with either trait, H1 indicates an association with only the first trait, H2 indicates an association with only the second trait, H3 suggests different causal variants associated with each trait, and H4 suggests that the same causal variant is associated with both traits. A posterior probability for H4 (PPH4) greater than 0.8 was considered strong evidence for colocalization, while PPH4 values greater than 0.5 indicated moderate colocalization.

#### Identification of pleiotropic loci and genes using PLACO

The pleiotropic analysis under the composite null hypothesis (PLACO) method was employed to identify SNP-level pleiotropy between autoimmune diseases and AN [18]. SNPs reaching an FDR-corrected P-value < 0.05 were considered pleiotropic variants and were retained for subsequent gene-level analysis. Nearby genes were mapped based on lead SNPs within each locus. To explore the biological functions of these pleiotropic loci, gene-set analyses were performed using the multi-marker analysis of genomic annotation (MAGMA) method [33].

A Bayesian colocalization analysis was conducted to further confirm the shared loci between autoimmune diseases and AN [34]. Genome-wide tissue-specific enrichment analysis was performed using 30 GTEx tissues [35] to examine the functional relevance of the identified pleiotropic loci. Gene Ontology (GO) and Kyoto Encyclopedia of Genes and Genomes (KEGG) pathway enrichment analyses were conducted through https://reactome.org/.

#### Causal association analysis

**Fig. 2:**
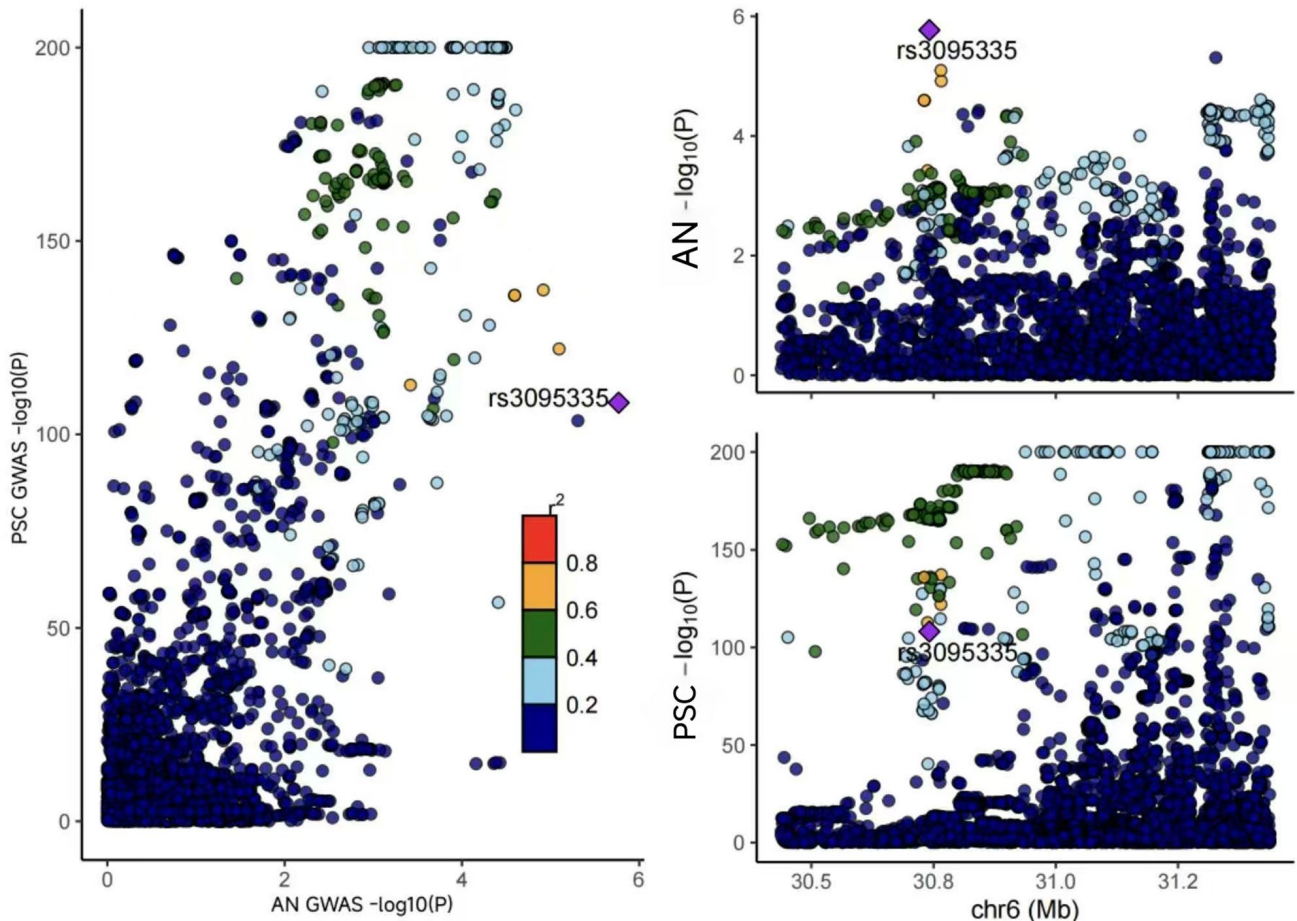
Colocalisation analysis results of primary sclerosing cholangitis with anorexia nervosa

A one-directional two-sample Mendelian randomization (MR) analysis was conducted to assess potential causal effects of autoimmune disorders on AN risk. Independent significant SNPs for autoimmune diseases (*P* < 5 × 10^− 8^) were extracted using the clumping procedure in PLINK 1.9, with an r² threshold of 0.001 and a 10,000 kb window [32]. The proportion of variance explained (PVE) and F-statistic (F > 10) were used to evaluate the strength of instrumental variables [36] (Fig. 2).

According to the causal diagram for MR shown in Fig. 1, the method must satisfy three assumptions: (1) IVs should be associated with the exposure; (2) IVs should influence the outcome only through the exposure; and (3) IVs should not be associated with any confounders. To ensure the robustness of causal inferences, eight different MR approaches were applied for each set of instrumental variables. These included inverse variance weighted (IVW), fixed-effects IVW, weighted median [37], weighted mode, MR pleiotropy residual sum and outlier (MR-PRESSO) [38], MR-Egger [39], simple mode, and simple median [40]. Heterogeneity across instrumental variables was assessed using Cochran’s Q-statistic [37, 41]. Horizontal pleiotropy was examined using the MR-Egger intercept and the MR-PRESSO global test [38, 39].

#### Software and packages

All statistical analyses were conducted using R (version 4.4.1). LDSC analysis was implemented using the LDSC software (v1.0.1) [14]. The PLACO method was applied using the PLACO package [18]while Bayesian colocalization analysis was performed using the coloc package (version 5.2.1) [34]. Gene and gene-set analyses were performed using MAGMA software [33]. GO analysis was conducted via the g: Profiler database, and KEGG pathway analysis was carried out using the clusterProfiler R package. Two-sample MR analysis was conducted using the MendelianRandomization package (version 0.9.0) [39], while MR-PRESSO analysis was implemented using the MR-PRESSO package (version 1.0) [38].

## Results

### Shared genetic architecture between autoimmune disorders and anorexia nervosa

We first evaluated the genetic correlation between autoimmune diseases and anorexia nervosa using linkage disequilibrium score regression (LDSC). The results are summarized in Table 1 and Additional file 1: Table S2. Three autoimmune diseases, including AOA, COA, and PSC, were found to be significantly genetically correlated with AN. Given this significant correlation, these three autoimmune diseases were selected for further analysis.

**Table 1:** Genetic correlation between autoimmune diseases and AN

Trait pairs	LDSC *r*_g_ (SE)	LDSC *P*
AN&PSC	0.208 (0.074)	0.005
AN&HT	− 0.054 (0.032)	0.092
AN&RA	− 0.081 (0.075)	0.283
AN&MS	0.090 (0.138)	0.515
AN&SS	− 0.123 (0.219)	0.572
AN&IBD	− 0.033 (0.062)	0.585
AN&T1D	− 0.019 (0.037)	0.599
AN&UC	− 0.029 (0.067)	0.665
AN&vitiligo	− 0.062 (0.151)	0.678
AN&SLE	− 0.056 (0.167)	0.734
AN&HD	0.018 (0.055)	0.744
AN&GD	− 0.007 (0.050)	0.886
AN&PBC	− 0.013 (0.117)	0.911
AN&CD	− 0.001 (0.038)	0.968
AN&COA	0.152 (0.023)	1.35 × *10*^–10^
AN&AOA	0.190 (0.027)	2.20 × *10*^–12^

LDSC linkage disequilibrium score regression, SE standard error, AN Anorexia nervosa, AOA adult-onset asthma, COA childhood-onset asthma, GD Grave’s disease, HD Hashimoto’s disease, HT hypothyroidism, PBC primary biliary cirrhosis, PSC primary sclerosing cholangitis, IBD inflammatory bowel disease, CD Crohn’s disease, UC ulcerative colitis, RA rheumatoid arthritis, MS multiple sclerosis, SS systemic sclerosis, SLE systemic lupus erythematosus, T1D type 1 diabetes

### Colocalization of autoimmune diseases with anorexia nervosa

Colocalization analysis was conducted to investigate whether autoimmune diseases share causal genetic variants with AN. The results are presented in Table 2. Among the analyzed trait pairs using the most significant lead SNP for each disease, only PSC showed suggestive colocalization with AN (PP.H4 = 0.854, Table 2). In a subsequent expanded analysis including all genome-wide significant SNPs (*P* < 5 × 10⁻⁸) for each autoimmune disease, a total of 12 loci exhibited strong colocalization evidence (PP.H4 > 0.8) with AN across multiple autoimmune disorders (e.g., RA, SLE, GD; see Additional file 1: Table S3). Additionally, several autoimmune diseases—particularly rheumatoid arthritis (RA), systemic lupus erythematosus (SLE), and Graves’ disease (GD)—exhibited possible colocalization signals (PP.H4 > 0.4) at multiple AN risk loci (Table 2).More specific colocalization results for autoimmune diseases and anorexia nervosa are presented in Table S3.

**Table 2:** Colocalization analysis between autoimmune diseases and AN

Trait pairs	PP.H0	PP.H1	PP.H2	PP.H3	PP.H4
AN&PSC	5.75 × *10*^–214^	2.04 × *10*^–2^	3.55 × *10*^–213^	0.125	0.854
AN&RA	4.41 × *10*^–9^	0.527	7.90 × *10*^–12^	4.73 × *10*^–4^	0.471
AN&SLE	0.061	0.495	1.10 × *10*^–4^	4.44 × *10*^–4^	0.442
AN&GD	1.21 × *10*^–44^	0.455	3.38 × *10*^–45^	0.125	0.418
AN&PBC	2.72 × *10*^–10^	0.706	4.88 × *10*^–13^	9.74 × *10*^–4^	0.292
AN&UC	0.582	0.234	0.001	2.23 × *10*^–4^	0.181
AN&IBD	1.12 × *10*^–9^	0.889	8.28 × *10*^–11^	0.0654	0.0449
AN&COA	0	0.933	0	0.039	0.027
AN&Vitiligo	0.137	0.785	0.010	0.057	0.010
AN&MS	2.05 × *10*^–61^	0.974	3.57 × *10*^–63^	0.016	0.008
AN&CD	6.87 × *10*^–102^	0.857	8.03 × *10*^–103^	0.1	0.004
AN&AOA	0	0.414	0	0.582	0.003
AN&SS	0.990	0.006	0.001	9.75 × *10*^–6^	0.001
AN&HT	4.15 × *10*^–166^	0.939	2.26 × *10*^–167^	0.051	0.001
AN&HD	1.74 × *10*^–38^	0.933	1.06 × *10*^–39^	0.056	0.001
AN&T1D	1.30 × *10*^–200^	0.927	8.80 × *10*^–202^	0.062	0.001

PP.H0-4 the posterior probability of H0-4, AN Anorexia Nervosa, AOA adult-onset asthma, COA childhood-onset asthma, GD Grave’s disease, HD Hashimoto’s disease, HT hypothyroidism, PBC primary biliary cirrhosis, PSC primary sclerosing cholangitis, IBD inflammatory bowel disease, CD Crohn’s disease, UC ulcerative colitis, RA rheumatoid arthritis, MS multiple sclerosis, SS systemic sclerosis, SLE systemic lupus erythematosus, T1D type 1 diabetes

### Pleiotropic loci and genes identified for multiple autoimmune disorders and anorexia nervosa

**Fig. 3:**
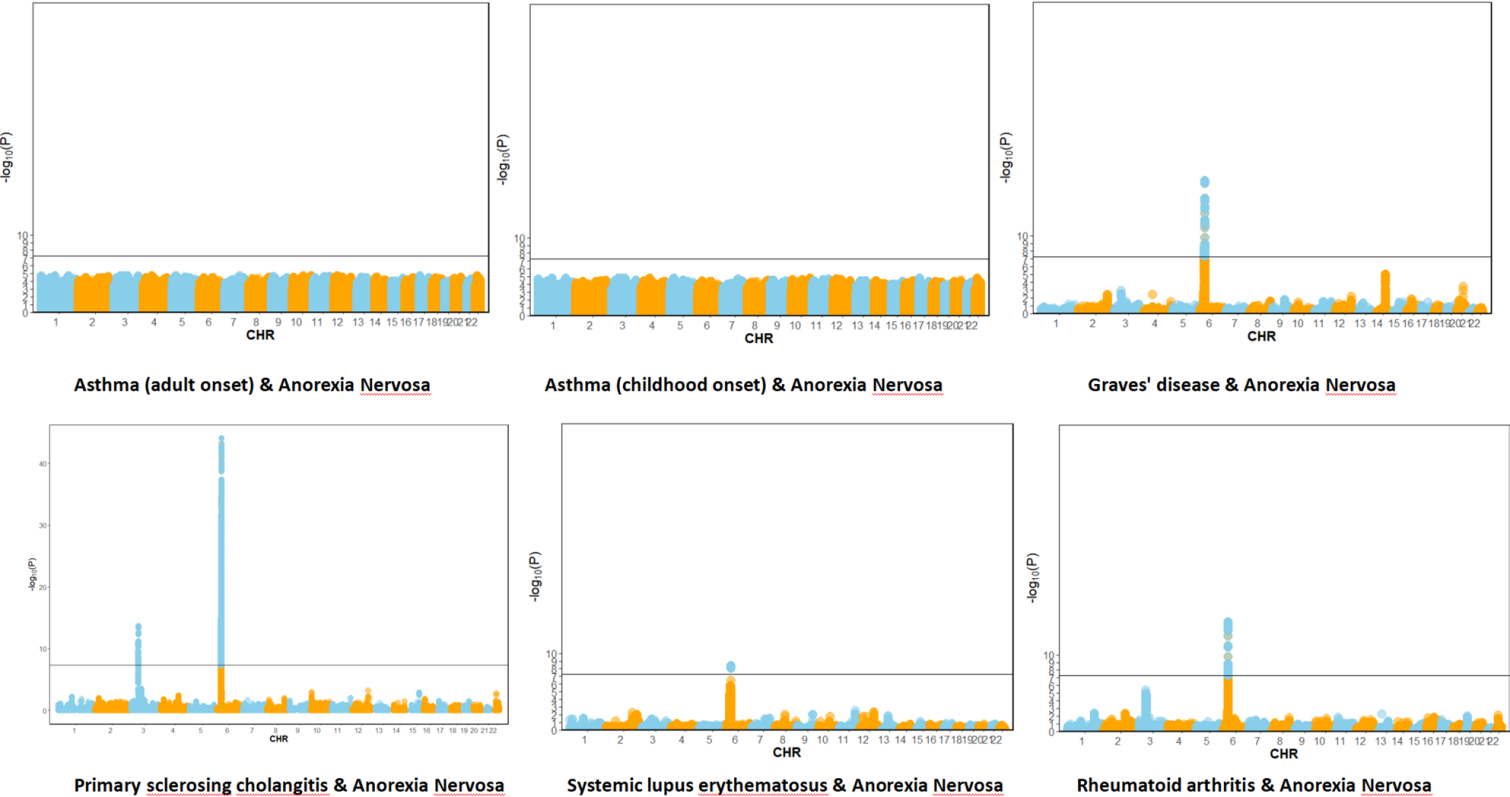
Manhattan plot of PLACO results for significant autoimmune disease associated with anorexia nervosa. The black line indicates that the -logP equals 7.3

Given the shared genetic architecture identified between autoimmune diseases and AN through LDSC and colocalization analyses, pleiotropy analysis was conducted using the PLACO method. The Manhattan plot illustrating PLACO results is shown in Fig. 3. The analysis revealed that genetic overlap between AN and autoimmune diseases, including RA, PSC, SLE, and GD, was primarily located on chromosomes 6 and 3. Pleiotropic SNPs with FDR-corrected P-values < 0.05 were selected for further investigation. The raw Placo analysis data for Autoimmune Disorders and Anorexia Nervosa can be found in Additional Files 2 and 3, and the detailed information of the 38 genome-wide significant pleiotropic genes identified via MAGMA is presented in Additional file 1: Table S4.

### Gene MAGMA analysis based on PLACO results

**Fig. 4:**
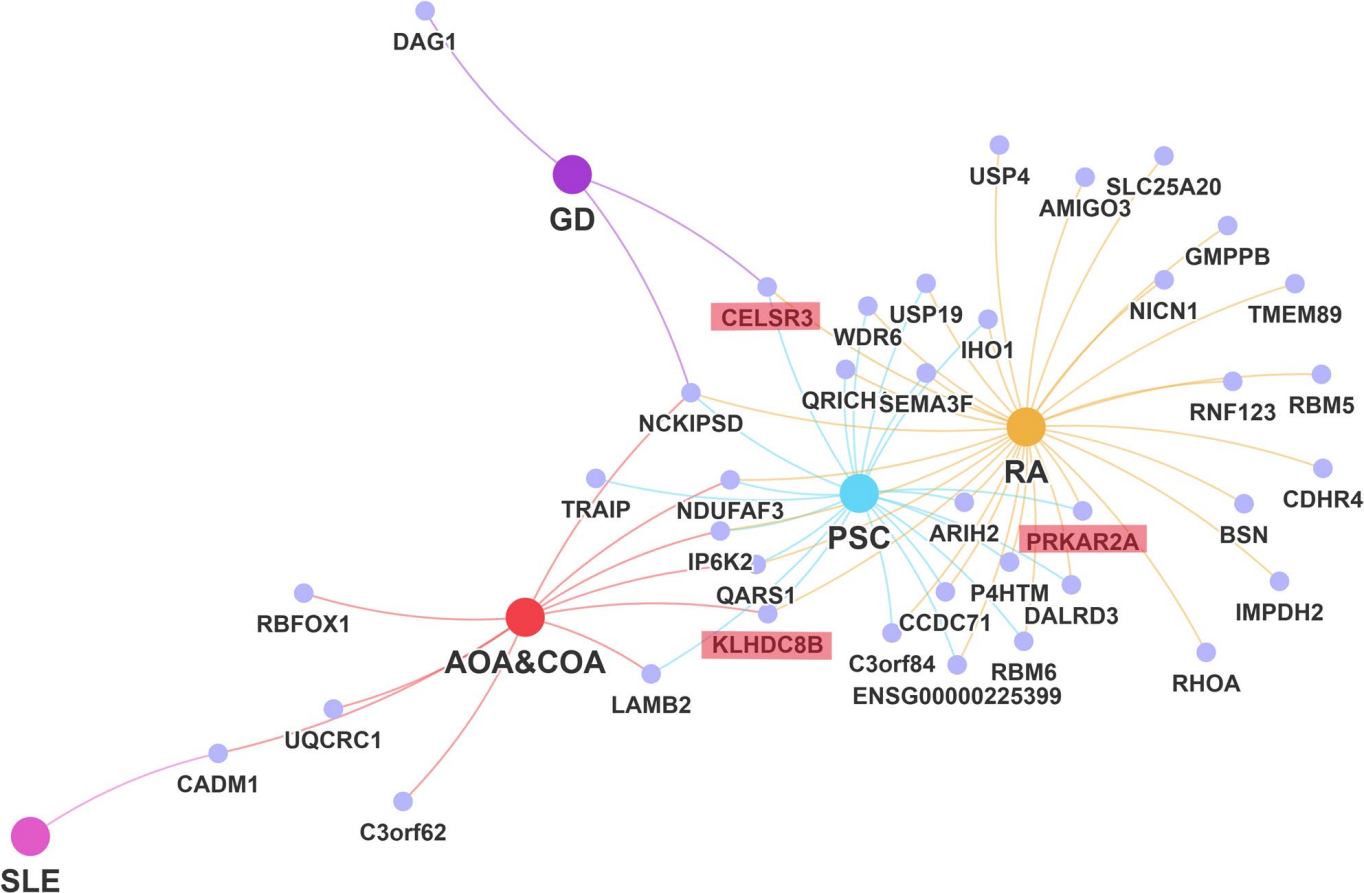
Network plot of gene magma analysis based on PLACO results of significant autoimmune diseases versus anorexia nervosa

Following the identification of pleiotropic loci via PLACO analysis, we employed a two-step procedure to ensure robustness and minimize false positives in subsequent gene-based analysis. First, genome-wide PLACO p-values for each AN–autoimmune disease pair were subjected to False Discovery Rate (FDR) correction using the Benjamini–Hochberg procedure. Only SNPs with an FDR-corrected p-value < 0.05 were considered statistically significant pleiotropic loci. Second, gene-based analysis was performed specifically on these significant pleiotropic SNPs using MAGMA. Genes with a corrected p-value less than 5 × 10^− 8^ were identified as pleiotropic genes (Fig. 4 and Additional file 1: Table S4).

The results indicated that AOA and COA shared the same pleiotropic loci. Notably, NCKIPSD was identified as a pleiotropic locus for four autoimmune diseases, excluding SLE. Additional genes, including CELSR3, NDUFAF3, IP6K2, QARS1, and KLHDC8B, showed evidence of pleiotropy across at least three autoimmune diseases. These genes were subsequently carried forward for colocalization analysis and gene enrichment studies to further investigate their genetic relationship with AN.

###  Colocalization analysis between genes and anorexia nervosa

**Fig. 5:**
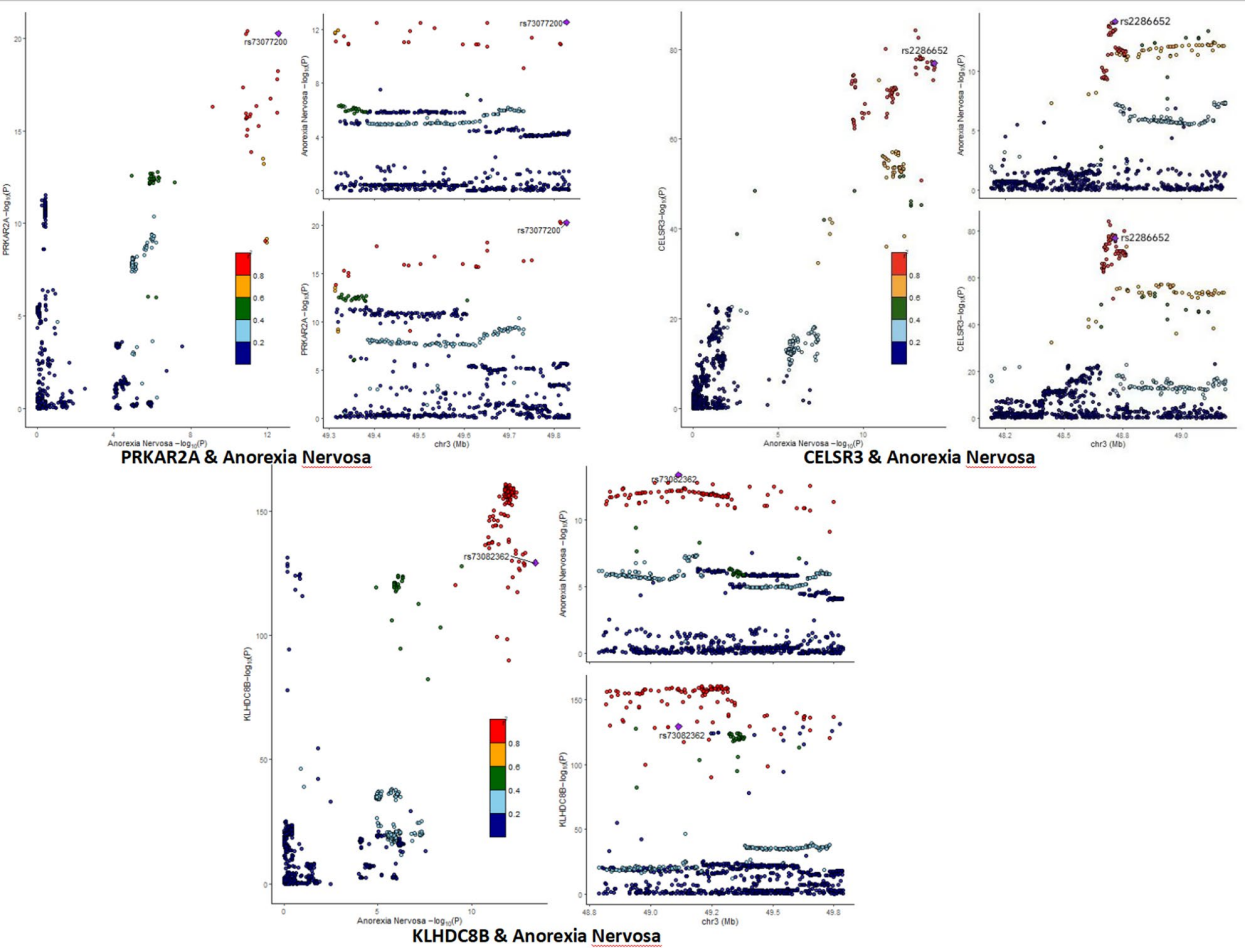
Results of colocalization analysis of significant genes with anorexia nervosa

Expression quantitative trait locus (eQTL) data for this analysis were obtained from the eQTL Catalogue via the IEU OpenGWAS platform, predominantly derived from blood tissues (e.g., peripheral blood, lymphoblastoid cell lines). To further examine gene-level colocalization with AN, we analyzed 30 genes that had significant cis-eQTLs (*p* < 5 × 10⁻⁸) available in this resource (Additional file 1: Table S5). Among these genes, PRKAR2A, CELSR3, and KLHDC8B demonstrated significant colocalization with AN, with PPH4 values greater than 0.75 (Fig. 5; Table 3). Interestingly, CELSR3 was identified as a pleiotropic gene for GD, PSC, and RA, while KLHDC8B was found to be shared between AOA, COA, RA, and PSC. Furthermore, PRKAR2A was identified in our study as a significant colocalized gene associated with PSC and AN (also a pleiotropic gene for RA, as shown in Table S4).

**Table 3:** Significant colocalization analysis between genes and AN

Gene symbol	PP.H0	PP.H1	PP.H2	PP.H3	PP.H4
PRKAR2A	2.68 × *10*^–24^	8.47 × *10*^–10^	4.72 × *10*^–17^	0.013	0.986
CELSR3	7.00 × *10*^–88^	9.20 × *10*^–11^	9.37 × *10*^–79^	0.122	0.878
KLHDC8B	4.79 × *10*^–163^	1.27 × *10*^–9^	6.03 × *10*^–155^	0.158	0.842

PP.H0-4 the posterior probability of H0-4

### Gene enrichment analysis

**Fig. 6:**
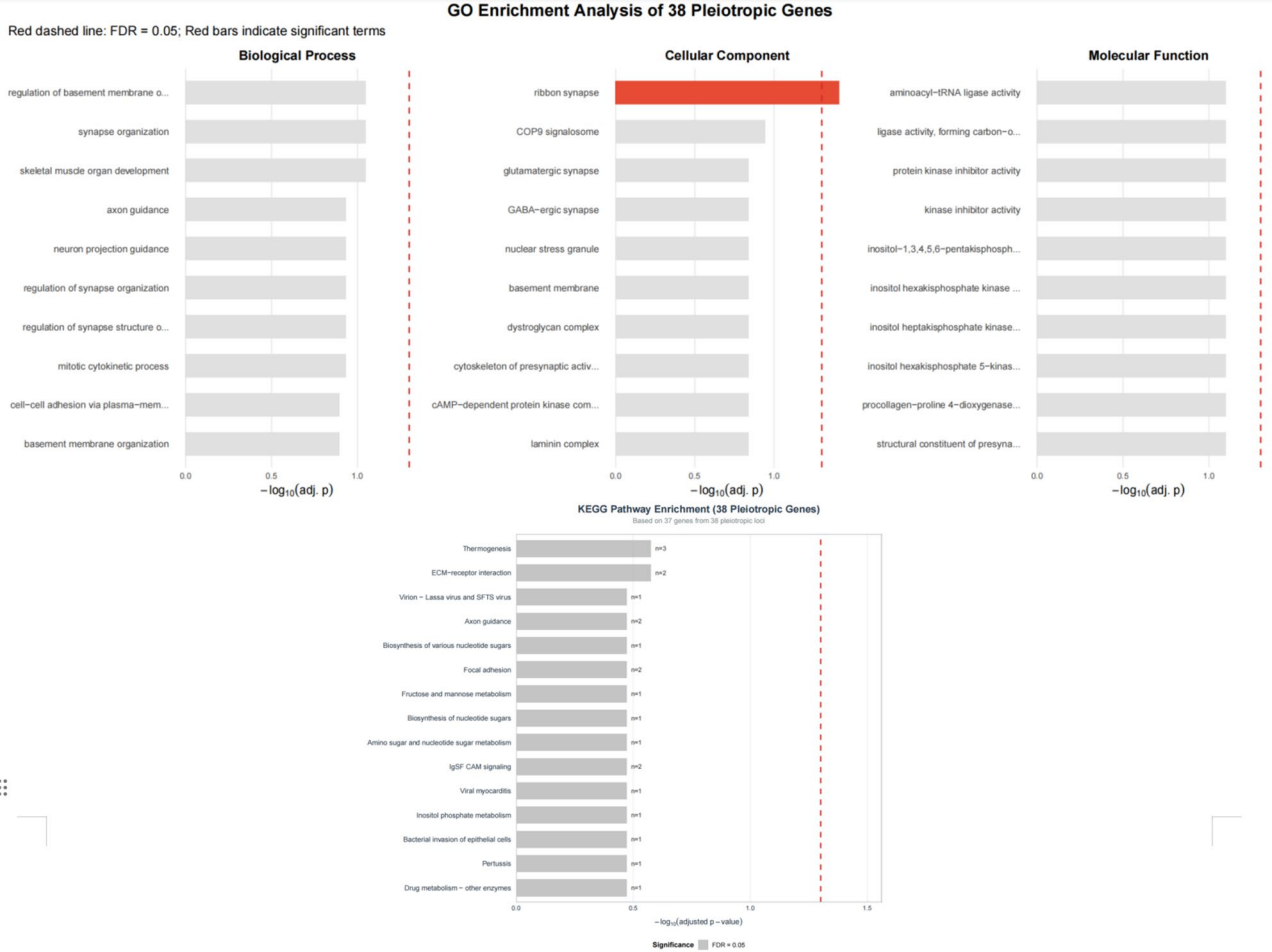
Results of gene GO and KEGG enrichment analysis

GO enrichment analysis revealed that among the identified genes, only the cell component term ‘ribbon synapse’ reached statistical significance after multiple testing correction (adjusted *p* = 0.019; Additional file 1: Table S6). KEGG pathway analysis indicated a nominal enrichment in thermogenesis-related pathways, though the adjusted p-value did not reach statistical significance (adjusted *p* = 0.15; Additional file 1: Table S7) (Fig. 6).

### MAGMA tissue analysis based on PLACO results

**Fig. 7:**
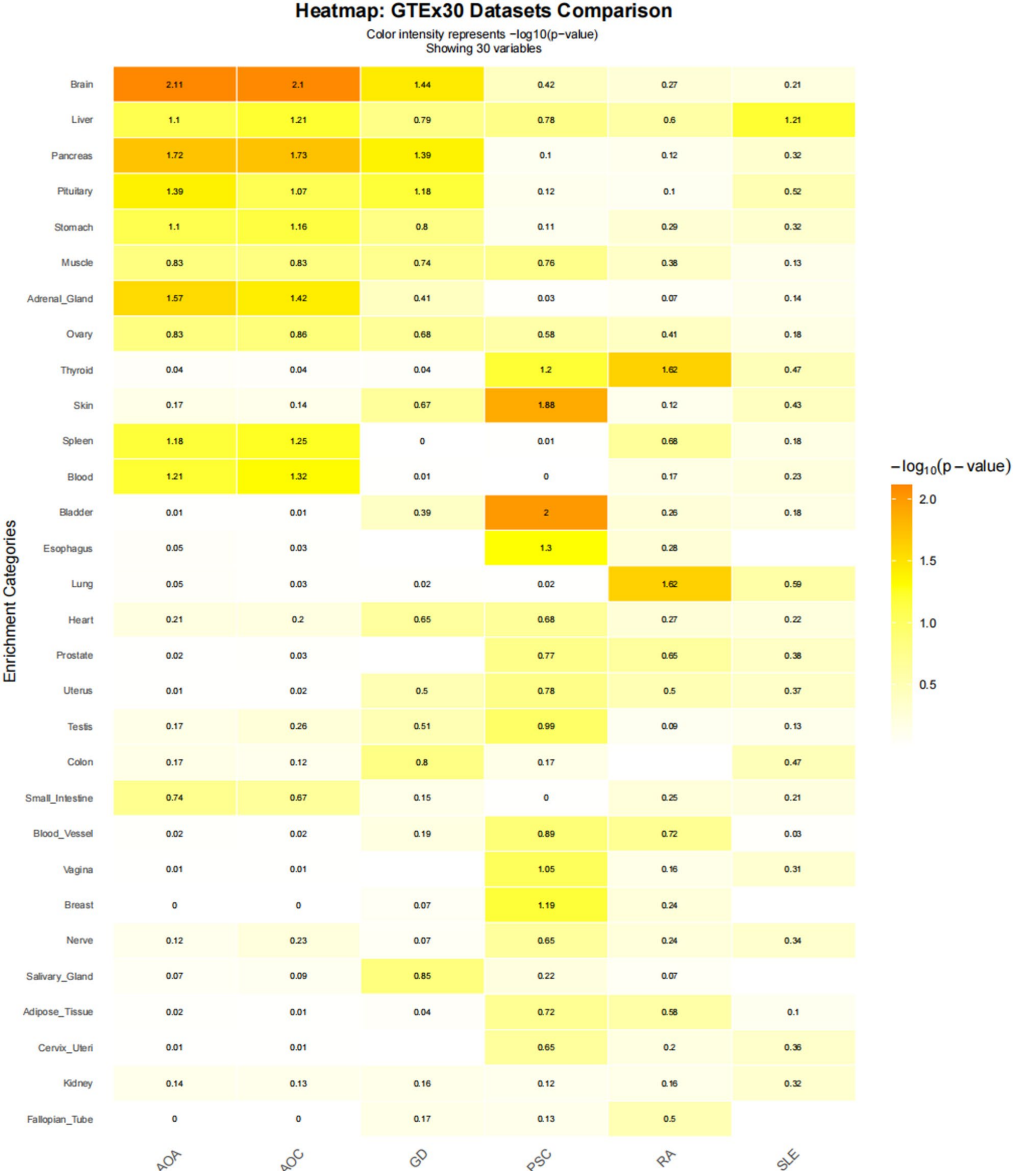
Results of magma 30 tissues based on the results of PLACO analysis

Tissue-specific enrichment analysis based on the significant loci identified in PLACO was performed using MAGMA (Fig. 7 and Additional file 1: Table S8). The results indicated that AOA, COA and GD-associated loci exhibited strong enrichment in brain tissues (FDR < 0.05). Notably, the pancreas showed nominally significant enrichment across all three disorders (AOA, COA, and GD). No significant enrichment was observed in the pituitary gland or stomach after multiple testing correction. Furthermore, PSC and RA-related loci were found to be nominally enriched in bladder (*p* = 0.010) and lung (*p* = 0.024) tissues, respectively, whereas SLE-related loci showed a trend of enrichment in liver tissue that did not reach nominal significance (*p* = 0.062).

### The causal relationship between autoimmune diseases and anorexia nervosa estimated by MR

**Fig. 8:**
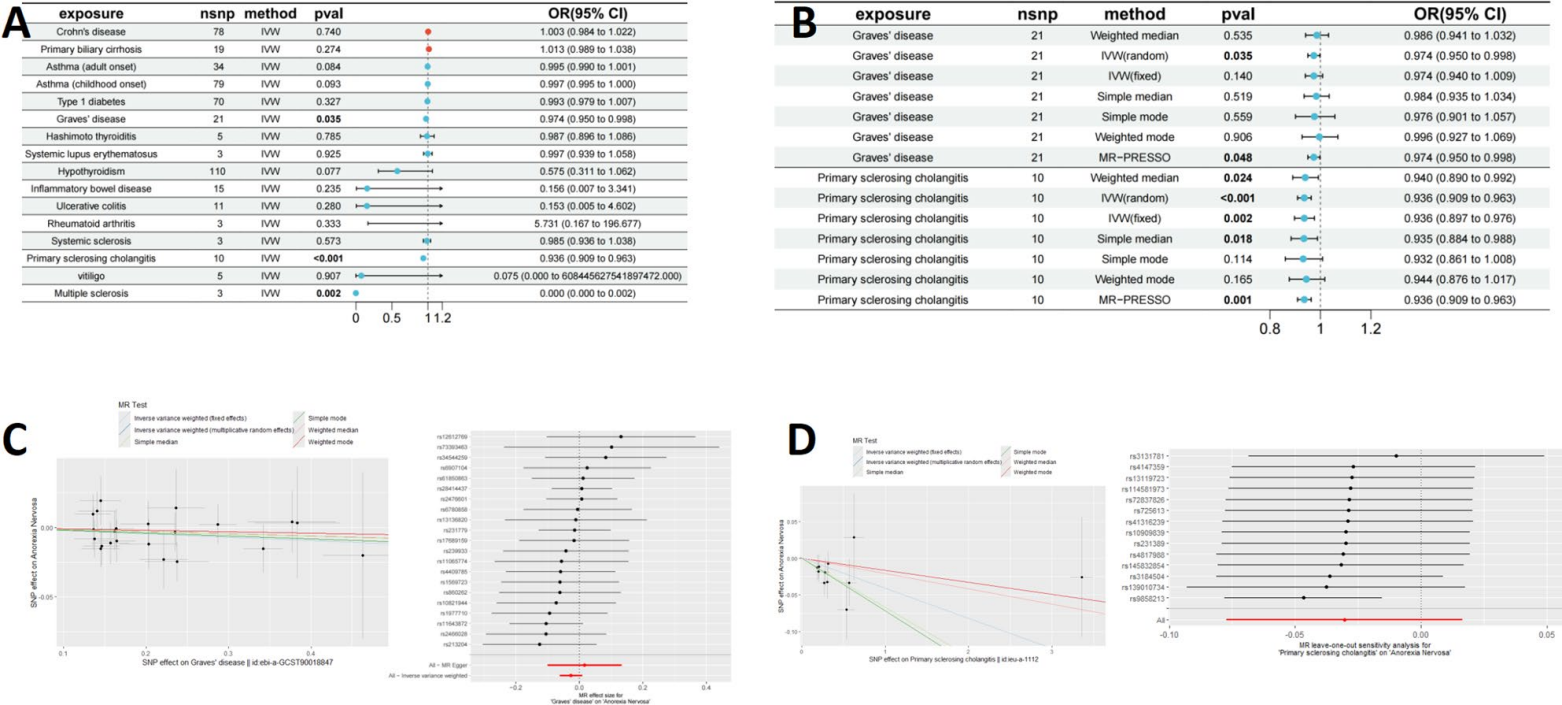
**A** The forest plot shows causal associations between autoimmune disorders and anorexia nervosa by using one-directional MR analysis. Causal effects were estimated by using IVW method. **B** Forest plot shows causal effects of GD and PSC on anorexia nervosa risk estimated by using different methods. **C** Scatter plot and leave-one-out analyse plot shows significant causal association between GD and anorexia nervosa risk. **D** Scatter plot and leave-one-out analyse plot shows significant causal association between PSC and anorexia nervosa risk

MR analysis was performed using the IVW method to estimate the causal relationship between autoimmune diseases and AN (Fig. 8 and Additional file 1: Table S10). The SNPs are shown in Additional file 1: Table S9. The results indicated a protective effect of PSC and GD on AN risk. Due to the limited number of SNPs available for MS, the confidence interval was too narrow, making it unsuitable for interpretation despite its P-value being below the significance threshold.

Specifically, a significant inverse causal effect was observed for PSC on AN, with an estimated effect size of OR = 0.936 (95% CI: 0.909–0.963, *P* = 6.85 × 10^− 6^), as determined using the IVW method. Similarly, GD onset exhibited a protective effect on AN risk (OR = 0.974, 95% CI: 0.950–0.998, *P* = 0.035). Results from additional MR methods were consistent with the IVW findings. The MR-Egger intercept and the MR-PRESSO global test ruled out the presence of horizontal pleiotropy (Additional file 1: Table S11-13). Furthermore, scatter plots and leave-one-out sensitivity analyses confirmed that the findings were not driven by outlier SNPs (Fig. 8C–D).

After Bonferroni correction, only the causal association between PSC and AN remained statistically significant (*P* = 6.85 × 10^− 6^ < 0.05/16). Reverse MR analysis was conducted to test for bidirectional causality, and no evidence supporting reverse causality was detected (Additional file 1: Table S14).

## Discussion

The study employed comprehensive genetic methodologies to investigate the genetic correlation between autoimmune disorders and AN. Pleiotropic loci were determined using cross-trait PLACO analysis, and pleiotropic genes were identified through the MAGMA method. Subsequently, key pathways involved were identified. Finally, comprehensive MR analysis and sensitivity analysis inferred potential causal effects of two specific autoimmune diseases (i.e., PSC and GD) on AN.

Through genetic correlation analysis, we observed significant genetic overlap between AN and three autoimmune diseases, namely AOA, COA, and PSC. In contrast, genetic correlations with GD, RA, and SLE were not statistically significant. We provided strong evidence for shared genetic mechanisms between PSC and AN, with MR analysis further supporting a protective effect of PSC against AN. Given the limited research on the PSC-AN relationship, our findings offer novel insights. Evidence suggests that ursodeoxycholic acid (UDCA) is widely used in PSC treatment [42]. A case report described a patient with citrullinemia (CD) who exhibited severe anorexia and weight loss, initially diagnosed as AN. After undergoing a low-carbohydrate diet and oral supplementation of arginine and UDCA, the patient’s condition gradually improved [43]. This suggests that PSC may exert a protective effect against AN via UDCA. However, as MR analysis leverages genetic instruments to minimize potential confounders, we propose that PSC itself, beyond the effects of UDCA, plays a crucial role in AN risk modulation.

Furthermore, we observed a significant protective effect of GD against AN, which appears contradictory to previous case reports. A study described two young female patients with comorbid AN and GD (aged 20 and 26 years, respectively), whose anorexic symptoms improved following partial thyroidectomy [44]. These cases suggest that endocrine factors related to thyroid function may influence AN pathogenesis. Our findings highlight the need for further investigations into the mechanistic relationship between GD and AN.

We identified a series of genetic risk loci associated with both autoimmune diseases and AN, with several loci observed across multiple phenotypic pairs, including CELSR3, KLHDC8B, NCKIPSD, NDUFAF3, IP6K2, and QARS1. Previous studies have demonstrated that these loci play critical roles in the development of both autoimmune diseases and AN. For instance, NCKIPSD has been identified as a potential therapeutic target for AN based on genetic analyses focusing on AN susceptibility mechanisms [45]. Additionally, NDUFAF3 has been proposed as a potential drug target for vitiligo [46]. A significant association has also been observed between rs11171739, a trans-B cell-specific regulatory variant within the 12q13.2 autoimmune disease locus, and the upstream region of IP6K2 [47]. Notably, polymorphisms within the intergenic region between IP6K2 and PRKAR2A, including the SVA-D polymorphism and poly-T short tandem repeats in the 3’UTR of FOXP1, may regulate nearby genes and influence AN susceptibility [45]. Moreover, a recent multi-omics study integrating transcriptomic and proteomic data identified PRKAR2A as a high-confidence risk gene for AN through probabilistic finemapping, showing a posterior inclusion probability (PIP) of 0.814 in GTEx tibial artery tissue, indicating strong evidence for a causal role in AN. In pathway analyses, PRKAR2A was statistically overrepresented in the “regulation of immune system process” gene set al.ongside other immune-related genes such as MST1, TREX1, and PROS1, further reinforcing the involvement of immune pathways in AN pathophysiology [48]. Furthermore, PRKAR2A was identified as a pleiotropic gene for PSC, RA, and AN.

A search of the GWAS catalog revealed that CELSR3 has been reported to be associated with multiple autoimmune diseases, including CD, IBD, and UC [26]. Additionally, CELSR3 and NCKIPSD have been linked to AN susceptibility [31, 49]. KLHDC8B has been implicated in Behçet’s disease [50], while IP6K2 has been associated with COA [51]. These findings provide further evidence for a shared genetic basis between autoimmune diseases and AN.

The shared genetic architecture observed in our study suggests common mechanisms linking autoimmune diseases and AN. The identified genes are involved in multiple biological pathways, including basement membrane organization, ribbon synapse composition, and aminoacyl-tRNA ligase activity. For AOA, COA, and GD, we observed significant polymorphic enrichment in brain tissues, a key region for appetite regulation and feeding behavior. These genes may exert their protective effects on AN by modulating neural projections within brain regions involved in appetite control. However, the precise mechanisms underlying this genetic interplay require further investigation.

Our study has several limitations that should be considered when interpreting the findings. First, as with similar genetic studies, we utilized summary-level GWAS data rather than individual-level datasets, which precludes stratified analyses based on factors such as age and detailed environmental covariates.

Second, and of particular relevance to the traits studied, is the potential for stratified bias due to differing sex distributions. Anorexia nervosa exhibits a strong female preponderance, while the 16 autoimmune diseases encompass a spectrum of sex ratios, from female-predominant (e.g., systemic lupus erythematosus) to male-predominant (e.g., ankylosing spondylitis, though not all were included). This heterogeneity raises the possibility that the observed genetic overlaps could be confounded or driven by sex-specific genetic effects. Our analyses employed sex-combined GWAS summary statistics, which cannot disentangle such effects. While methods like LDSC are robust to many confounding structures, they cannot fully account for complex sex-by-genotype interactions. The inability to perform sex-stratified analyses due to data availability represents a major limitation and a crucial direction for future research.

Third, the sample sizes for some of the GWAS datasets, including that for AN, were relatively limited. Therefore, caution is warranted when interpreting the effects of specific loci, and our results require replication in larger cohorts.

Fourth, our study was restricted to individuals of European ancestry to minimize population stratification. This limits the generalizability of our findings to other ancestral populations, and future cross-ancestry studies are essential.

Given these limitations, particularly the constraints on statistical power and the inability to explore sex-stratified effects, the conclusions drawn from this study should be considered preliminary and interpreted with caution.

## Conclusions

Our study elucidates the intricate relationship between autoimmune diseases, particularly AOA, COA, PSC, GD, RA, and SLE, and AN. We identified pleiotropic risk genes shared across these conditions, including PRKAR2A, CELSR3, and KLHDC8B, suggesting the presence of common underlying mechanisms. Furthermore, our findings provide evidence of a causal relationship between autoimmune diseases (GD and PSC) and AN.

## Supplementary Information

Below is the link to the electronic supplementary material.

Supplementary Material 1.

Supplementary Material 2.

Supplementary Material 3.

## Data Availability

All data generated or analyzed during this study are included in this published article.
